# Supplementary material for: MetaRibo-Seq measures translation in microbiomes
Source: Nat Commun. 2020 Jun 29;11:3268. doi: 10.1038/s41467-020-17081-z (PMC7324362; doi:10.1038/s41467-020-17081-z)
Supplement: Supplementary file 10 — Supplementary Data 7 [file 41467_2020_17081_MOESM10_ESM.zip › File2/Confidence_High_Taxonomy/91739_out.krona.html]

Javascript must be enabled to view this page.

members
magnitude
magnitudeUnassigned
count
unassigned
taxon
rank

91739\_out

3

3
2
superkingdom

phylum
1239
3

class
186801
3

186802
3
order

186803
3
family

3
712976

SRS022143\_contig\_number\_20885SRS101439\_contig\_number\_contig-100\_9832.149268SRS149690\_contig\_number\_contig-100\_33880.33881
species
